# Fast Detection of Unexpected Sound Intensity Decrements as Revealed by Human Evoked Potentials

**DOI:** 10.1371/journal.pone.0028522

**Published:** 2011-12-06

**Authors:** Heike Althen, Sabine Grimm, Carles Escera

**Affiliations:** 1 Institute for Brain, Cognition and Behavior (IR3C), University of Barcelona, Barcelona, Catalonia, Spain; 2 Cognitive Neuroscience Research Group, Department of Psychiatry and Clinical Psychobiology, University of Barcelona, Barcelona, Catalonia, Spain; University of Regensburg, Germany

## Abstract

The detection of deviant sounds is a crucial function of the auditory system and is reflected by the automatically elicited mismatch negativity (MMN), an auditory evoked potential at 100 to 250 ms from stimulus onset. It has recently been shown that rarely occurring frequency and location deviants in an oddball paradigm trigger a more negative response than standard sounds at very early latencies in the middle latency response of the human auditory evoked potential. This fast and early ability of the auditory system is corroborated by the finding of neurons in the animal auditory cortex and subcortical structures, which restore their adapted responsiveness to standard sounds, when a rare change in a sound feature occurs. In this study, we investigated whether the detection of intensity deviants is also reflected at shorter latencies than those of the MMN. Auditory evoked potentials in response to click sounds were analyzed regarding the auditory brain stem response, the middle latency response (MLR) and the MMN. Rare stimuli with a lower intensity level than standard stimuli elicited (in addition to an MMN) a more negative potential in the MLR at the transition from the Na to the Pa component at circa 24 ms from stimulus onset. This finding, together with the studies about frequency and location changes, suggests that the early automatic detection of deviant sounds in an oddball paradigm is a general property of the auditory system.

## Introduction

The automatic detection of deviant or contextual novel stimuli is a crucial function of the auditory system, as it can trigger an attention switch to unexpected events (for a review, see [1]). It is reflected by the auditory evoked potential (AEP) called mismatch negativity (MMN; [2]), a negative deflection between 100 and 250 ms after stimulus onset with sources in auditory and prefrontal cortex areas [3]–[4] that is elicited by rare regularity-violating stimuli, which occur amongst a regular sound pattern. MMN is the most prominent component reflecting auditory deviance detection in humans. Yet, based on animal research, it has been proposed that the detection of deviant stimuli is a multi-stage process [5] that begins at early latencies of about 20 ms [6]–[7] and extends over auditory areas from the IC to the cortex (for a review, see [8]).

This hypothesis is supported by several recent studies that give evidence of a deviance-related modulation in the human middle latency response (MLR). According to Grimm et al. [9], frequency deviants of a controlled oddball paradigm elicit a more negative response than standard sounds at circa 40 ms after stimulus onset (Nb component). A still earlier deviance-related effect in the MLR was found by Slabu et al. [10]. They report an enhanced Pa component in response to band-pass-filtered broadband noise deviants at latencies of circa 30 ms. Moreover, the detection of location deviants has been shown to modulate the Na component of the MLR at approximately 25 ms after change onset [11]–[12]. This data suggest that deviance-related modulations at early latencies of the AEP occur for sound changes in frequency and location. However, whether this generalizes to other sound features, like intensity or duration, has not been investigated yet.

Furthermore encoding mechanisms of sound probabilities are evident at the cellular level at comparably early latencies in the form of a stimulus-specific adaptation (SSA) to repetitive stimuli; that is, a strong decrease of neuronal response and a sudden restoration of firing rates when deviant sounds occur. This phenomenon has been found in the mammalian inferior colliculus [7], [13], the thalamic medial geniculate body and reticular nucleus [6], [14], the auditory cortex [15]–[18], the avian external nucleus of the inferior colliculus, as well as in the avian homolog to the mammalian superior colliculus and frontal eye fields [19]. It is still a matter of discussion how those novelty-sensitive auditory neurons correspond to the cortical MMN. The MLR reflects the auditory evoked activity in the auditory cortex [20]–[21], and possibly also in the medial geniculate body of the thalamus [22]–[23] between circa 12 and 70 ms after stimulus onset. Therefore, the MLR facilitates the comprehension of the relation between SSA at the cellular level and the emergence of the scalp-recorded MMN, as shown by recent studies in humans mentioned above, in which the analysis of the MLR points to an earlier detection of frequency and location deviants than reflected by the MMN.

In this study, we aim at investigating whether the processing of deviant sound intensities becomes evident by modulations in the MLR or even in the auditory brainstem response (ABR) of the human AEP. This is based on the fact that, on the one hand, probabilities of stimulus intensity levels are encoded in terms of SSA at the neuronal level [16], [19], [24]; and on the other hand, that the MMN is elicited in response to stimuli with rare intensity increments and decrements [2], [25].

## Materials and Methods

### Ethics Statement

Participants gave informed written consent before the experiment. The experimental protocol was approved by the Ethical Committee of the University of Barcelona and was in accordance with the Code of Ethics of the World Medical Association (Declaration of Helsinki).

### Participants

Twenty-eight young, normal-hearing subjects (18–33 years, 14 females) participated in the experiment for payment (€6 per hour). None of them reported any neurological or psychiatric disorders or any treatment with psychotropic drugs. All participants had a hearing threshold below a peak-equivalent sound intensity of 30 dB SPL (group average = 22 dB peSPL) for click tones with a duration of 100 µs. The electroencephalogram (EEG) data of five participants had to be excluded from analysis due to a high number of artifacts.

### Experimental design and procedure

**Figure 1:**
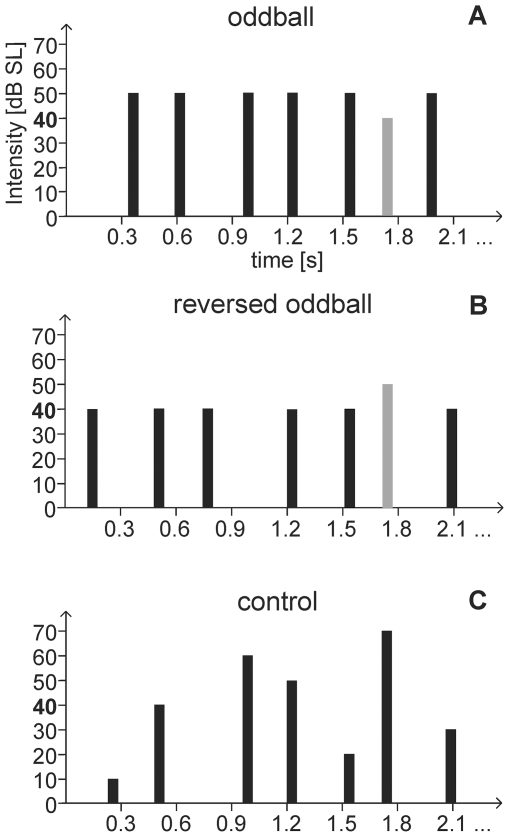
The three stimulation conditions. Stimuli were clicks of 100 µs duration, presented binaurally through headphones. The onset-to-onset interval varied randomly in 8-ms steps between 256 and 344 ms, with a mean of 300 ms **A.** In the oddball blocks, two types of stimuli were presented randomly. Standard stimuli, indicated in black, were presented with an intensity of 50 dB SL and a probability of 6/7. Deviant stimuli, indicated in grey, were presented with an intensity of 40 dB SL and a probability of 1/7. **B.** In the reversed oddball blocks, the intensities of standards and deviants were exchanged. Probabilities and colors are as in A. **C.** In the control blocks seven different stimuli with intensities of 10, 20, 30, 40, 50, 60 or 70 dB SL, each with a probability of 1/7 were presented randomly.

Participants were sitting comfortably in an electrically shielded and sound-attenuated room. They were asked to relax, to concentrate on a silent movie with subtitles and to ignore the sounds. Click tones of 100 µs duration were presented binaurally through headphones with an onset-to-onset interval varying randomly in 8-ms steps between 256 and 344 ms (mean = 300 ms). Sound intensities were presented above the individual hearing threshold (sensation level, dB SL) which was detected by means of an audiometry using the same stimuli as in the experiment. Sound presentation was controlled using the software MATLAB (Release 14) and the Psychophysics Toolbox extensions [26]. Stimuli were presented in three different auditory sequences: the oddball, reversed oddball and control condition (Fig. 1). In the oddball condition, stimuli were either standard sounds with an intensity level of 50 dB SL and a presentation probability of 6/7 or rare deviant sounds with a presentation probability of 1/7 and an intensity level of 40 dB SL. In the reversed condition, the presentation probabilities were the same as in the oddball condition, but the intensity levels of the stimulus types were swapped so that the standard stimuli were presented at an intensity level of 40 dB SL and the deviants at an intensity level of 50 dB SL (standard stimuli of this reversed condition will from now on be referred to as “standards”). This condition was included in order to compare AEPs to the same physical stimuli while holding different contextual roles. It should be noticed the outmost importance to control for physical differences of the stimuli, because especially at short latencies, the AEP is very sensitive to stimulus properties. This is demonstrated by the AEPs in response to the control stimuli. The latency and amplitude of the Wave V (ABR) and Na component (MLR) change systematically with stimulation intensity (see the results section below). Comparing AEPs in response to deviant and standard stimuli having different intensities could lead to mixing deviance-related modulations of the AEPs with signal differences due to the physical differences of the stimuli. An additional control condition served to control for the refractoriness-based explanation of deviance-related effects ([25], [27]; cf.[28]). It consisted of seven different stimuli with the intensities 10, 20, 30, 40, 50, 60 and 70 dB SL, each occurring with a probability of 1/7. Stimuli were presented randomly with the restriction that a deviant was preceded by at least two standards and a control stimulus was followed by at least two control stimuli of a different intensity. The three conditions were subdivided into 18, approx. 5.5 min. lasting, blocks. Blocks of the oddball and control conditions were presented alternating whereas the reversed condition was applied in the first and the last block. In total, standards, deviants and the different control stimuli were presented 1,248 times each.

### EEG acquisition

The EEG was recorded continuously from seven scalp electrodes (Ag/AgCl). An additional electrode was placed on the tip of the nose to analyze the MMN. Recording positions of the scalp electrodes were Cz, FCz, Fz, FC3, FC4, PO7, PO8, mounted according to the 10–20 system using an elastic cap (64-channel Quik-Cap, NeuroMedical Supplies, Compumedics, Charlotte, NC). Additionally, the eye movements were controlled with two bipolar electrodes placed above and below the left eye (vertical electrooculogram) and two electrodes placed at the outer canthi of each eye (horizontal electrooculogram). The recorded signal was referenced to linked electrodes placed on the earlobes. Impedances were kept below 5 kΩ. The amplified signal (SynAmpsRT, NeuroScan, Compumedics, Charlotte, NC) was online bandpass-filtered from 0.05 to 1500 Hz and digitized with a sampling rate of 20 kHz using the software Scan 4.4 (NeuroScan, Compumedics, Charlotte, NC).

### AEP analysis

For AEP analysis in the long latency range (LLR) and the MLR, the EEG-data was down-sampled to 2000 Hz. For offline filtering, bandpass filters ranging from 1.5 to 30 Hz for the LLR, from 15–250 Hz for the MLR, and from 100–1500 Hz for the ABR were applied. The LLR data was re-referenced to the nose. Thereafter, the AEPs to deviants, standards and controls were averaged separately in epochs ranging from −100 to 300 ms for the LLR, −50 to 100 ms for the MLR, and −30 ms to 20 ms for the ABR. Averaged epochs included a pre-stimulus baseline with duration of 100 ms, 50 ms and 30 ms respectively. Trials including peak-to-peak amplitudes exceeding 80 µV (LLR), 50 µV (MLR) or 35 µV (ABR) were automatically rejected. AEPs elicited by the control stimuli were analyzed in the MLR and ABR ranges. As the Wave V of the ABR in response to control stimuli of 40 dB SL was maximal at the FCz electrode and the Na component at Fz, AEPs at these electrodes were taken for statistical analyses and illustration. Peak amplitudes and latencies of Wave V and the Na component revealed a clear dependence on stimulation intensity. Therefore, linear regression analyses were calculated for peak amplitudes and latencies of those components. Moreover, peak amplitudes and latencies, elicited by adjacent control stimuli (e.g. control 20 dB SL and control 30 dB SL), were tested for differences with Wilcoxon-Tests or paired samples two-tailed Student's t-tests. With the purpose of disclosing deviance-related changes in the AEP, the responses to deviants, standards and control stimuli of 40 dB SL were compared. Consequently, all three stimulus types were physically equal and presented at the same intensity level. To evaluate the signal of the components Wave V, Na and Pa (Nb and Pb components were not elicited for stimulation at 40 dB SL), repeated measures analysis of variance (ANOVAs) were calculated on mean voltages of a time window centered on the grand-average peak. For Wave V, a 2-ms time window from 7 to 9 ms and for the Na and Pa component 10-ms time windows from 14 to 24 ms and from 26 to 36 ms, respectively, were used. The ANOVAs included the factors Comparison (standard and deviant, standard and control, deviant and control, respectively) and electrode (Fz, FCz, Cz, FC3 and FC4). Peak latencies of the Wave V and the Na component were tested at the electrodes FCz and Fz, respectively, for differences regarding the stimulus type (Wilcoxon's signed-rank test).

Additionally, difference waveforms of the response to the three stimulus types were plotted and screened visually for deflections. In the MLR, standard, deviant and control mean voltages of a 6-ms time window centered on the peak of the largest deflections of the deviant-standard difference waveform (21 to 27 ms) were compared. As the difference curve for deviants and controls peaked at 20 ms after stimulus onset, additionally the deviant and the control responses in a time window centered on the deviant-control difference waveform (17 to 23 ms) were compared. The mean amplitudes were tested for differences by repeated measures ANOVAs with the factors Comparison (standard and deviant, standard and control, deviant and control, respectively) and electrode (Fz, FCz, Cz, FC3 and FC4). In the LLR, mean voltages of the time window 158–188 ms centered on the deviant-standard difference wave and of the time window 163–193 ms centered on the deviant-control difference wave in the typical time range of MMN (100–250 ms) were tested for differences at the electrode FCz using paired samples two-tailed Student's t-tests. Paired samples two-tailed Student's t-tests and Wilcoxon's signed-rank tests were corrected using a Bonferroni correction for multiple comparisons. Results were considered significant when p<.05.

## Results

The number of individual trials per stimulus type after artifact rejection exceeded 900 trials for the ABR, 990 for the MLR, and 350 for the LLR. Click stimuli elicited a robust ABR, which depended linearly in amplitude and latency on the stimulation level, but not on the contextual novelty of a stimulus. For high stimulation levels, the four principle components of the MLR were clearly elicited. Strikingly, deviant stimuli in the oddball condition elicited a more negative response than standard stimuli at around 24 ms after stimulus onset, corresponding to the transition from the Na component to the Pa component. Moreover, the standard Na component was reduced compared to the response to the same physical control stimulus.

### Control condition

**Figure 2:**
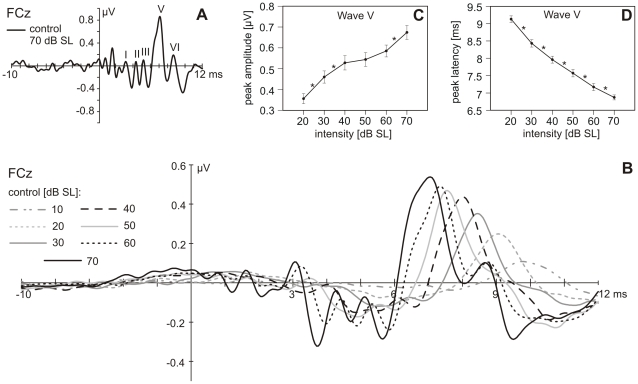
ABRs to the control stimuli. **A.** Single-subject recording to control stimuli of 70 dB SL at FCz. ABR components are labeled with Roman numerals. **B.** Grand-average response (N = 23) to the control stimuli of 10 to 70 dB SL at FCz. **C, D.** Mean Wave V peak amplitudes (**C**) and latencies (**D**) at FCz in response to the control stimuli presented at 20 to 70 dB SL. Error bars indicate +/- 1 standard error. Significant differences between adjacent control stimuli are indicated with an asterisk (p<0.05).

Wave V, the most prominent component of the ABR, could be identified at the single-subject level from a stimulus level of 10 to 20 db SL on. The subsequent Wave VI and the preceding Waves I, II, and III were pronounced for moderate to high stimulus levels (Fig. 2A). The peak amplitude of Wave V for stimulation from 20 to 70 dB SL displayed a positive linear relationship to the stimulation level (Fig. 2B,C), as revealed by a linear regression analysis (R^2^  = .307, β  = .555, t(136) = 7.771, p<.001). Comparison of peak amplitudes of Wave V elicited by adjacent stimulation intensities resulted in statistically significant differences for stimulation with 20 and 30 dB SL (z = −4.045, p<.001), 30 and 40 dB SL (z = −3.194, p<.01) and 60 and 70 dB SL (z = −3.771, p<.001; Fig. 2C). The peak latency of Wave V also depended on the stimulation level, being negatively related to stimulus intensity, that is, it decreased with increasing stimulation level (Fig. 2B,D; R^2^ = .722, β = −.849, t(136) = −18.771, p<.001). This relationship was also reflected in significantly different peak latencies in response to adjacent stimulation levels (Fig. 2D; 20/30 dB SL: *z* = −4.0, p<.001; 30/40 dB SL: *z* = −3.317, p<.01; 40/50 dB SL: *z* = −3.0, p<.01; 50/60 dB SL: *z* = −3.0, p<.01; 60/70 dB SL: *z* = −2.646, p<.01).

**Figure 3:**
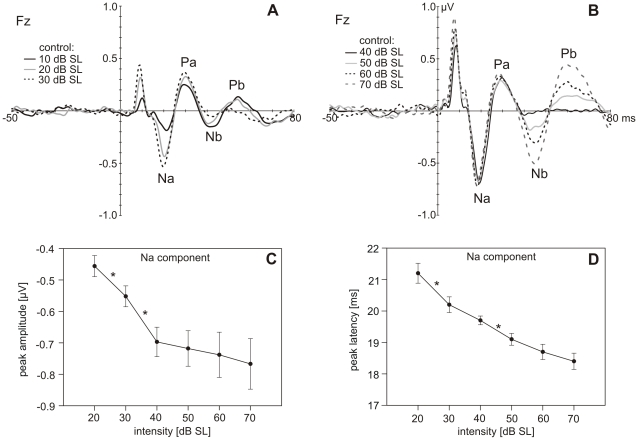
MLRs to the control stimuli. **A, B.** Grand-average AEP (N = 23) filtered for the MLR in response to control stimuli of 10 to 30 dB SL **(A)** and 40 to 70 dB SL **(B)** at Fz. **C,D.** Mean peak amplitudes (**C**) and latencies (**D**) of the Na component in response to the control stimuli of 20 to 70 dB SL at the electrode Fz (N = 18). Error bars indicate +/- 1 standard error. Significant differences between adjacent control stimuli are indicated with an asterisk (p<0.05).

In the MLR, the four typical components Na, Pa, Nb and Pb, were obtained (Fig. 3A,B). In Table 1, mean amplitudes, mean latencies as well as the latency range of the MLR components for stimulation with 70 dB SL are given. Whereas the Na and Pa components were elicited for most subjects already at lower intensities, the Nb and Pb components where measurable only for a part of the group when stimulating with low intensities. The Na amplitude and latency depended linearly on the stimulation intensity (Fig. 3C,D). Linear regression analysis revealed an amplitude increase (Fig. 3C; R^2^ = .161, β = −.402, t(106) = −4.518 p<.001) and latency decrease (Fig. 3D; R^2^ = .457, β = −.676, t(106) =  −9.441, p<.001) with increasing stimulation intensity. Na mean amplitudes for stimulation at 20 and 30 dB SL (t(17) = 3.623, p<.05) as well as for stimulation at 30 and 40 dB SL (t(17) = 3.381, p<.05) differed significantly from each other (Fig. 3C). Significant latency differences were observed for stimulation at 20 and 30 dB SL (z = −3.116, p<.05) as well as for stimulation at 40 and 50 dB SL (Fig. 3D; z = −3.051, p<.05).

**Table 1:** Mean latencies, latency ranges, and mean amplitudes of the MLR components Na, Pa, Nb and Pb, elicited by the control stimulus presented at an intensity of 70 dB SL. N = 18.

MLR components	Na	Pa	Nb	Pb
Mean latency [ms] (SEM)	18.4 (.26)	30.0 (.87)	44.9 (1.39)	61.94 (1.56)
Latency range [ms]	16–20	24–37	34–59	53–76
Mean amplitude [ µV] (SEM)	−.77 (.08)	.60 (.07)	−.81 (.07)	.86 (.08)

Standard errors of mean (SEM) are given in parentheses.

### Deviance-related effects

**Figure 4:**
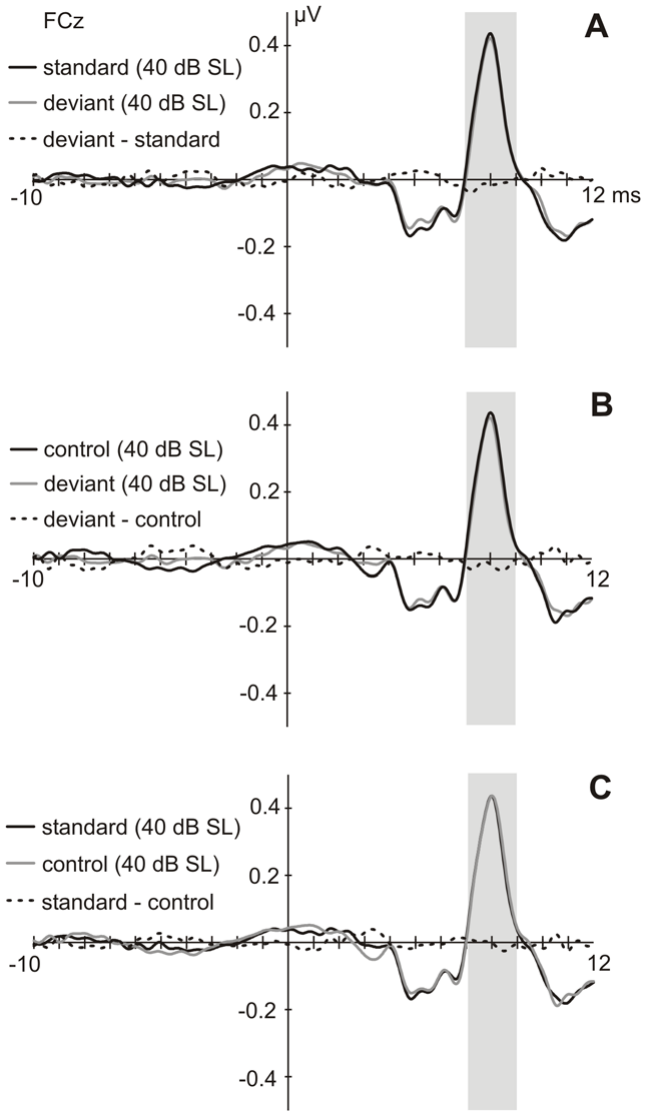
ABRs to standards, deviants and controls. Grand-average response (N = 23) to deviants and standards **(A)**, deviants and controls **(B)** and standards and controls **(C)** at FCz. The grey shaded bars denote the time window of the mean amplitudes used for statistics.

Peak latencies of Wave V elicited by deviant, standard and control stimuli were tested for differences at FCz. Mean amplitudes were tested for differences from 7 to 9 ms after stimulus onset at the electrodes Fz, FCz, Cz, FC3 and FC4. Repeated measures ANOVAs did not reveal any effect of stimulus type (Fig. 4A–C).

**Figure 5:**
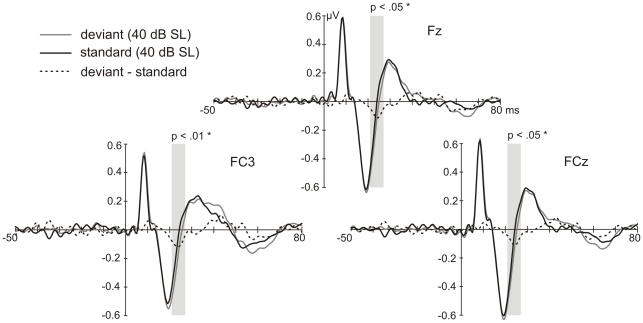
Deviance-related changes in the MLR. Grand-average response (N = 23) at FCz, FC3 and Fz elicited by deviants and standards. The grey shaded fields mark the time window of the mean amplitudes used for statistics. The difference waveforms reveal a negative displacement of the response to deviants compared to the one to standards.

**Figure 6:**
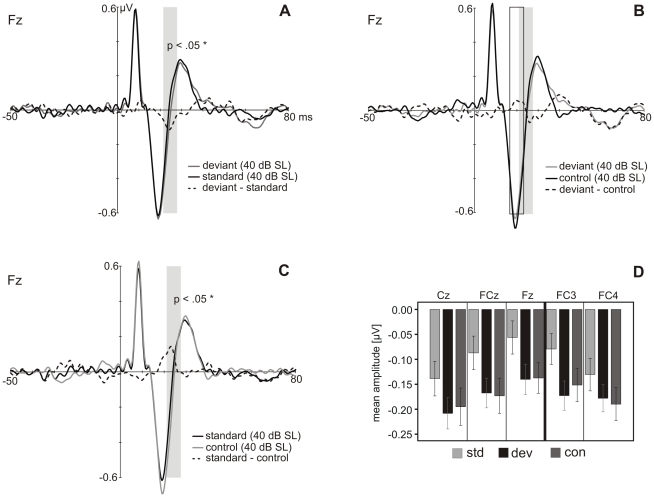
MLRs to the same physical stimulus presented in different conditions. **A**−**C.** Grand-average MLR responses (N = 23) to deviants, standards and controls at Fz. The grey shaded areas and the framed area mark the time windows of the mean amplitudes used for statistics. **D.** Mean amplitudes from 21 to 27 ms after stimulus onset (grey shaded field in A−C) in response to deviant (dev), standard (std) and control (con) at Cz, FCz, Fz, FC3 and FC4.

In the MLR, mean amplitudes of the Na and Pa components were tested for deviance-related modulations at the electrodes Fz, FCz, Cz, FC3 and FC4. No significant differences for the stimulus type were revealed. Visual inspection of the waveforms showed that the response to deviants and standards diverged at the transition from the Na to the Pa component (Fig. 5 and 6A,D), which became visible in the difference waveform (deviant-standard), peaking at 24 ms after stimulus onset (see Fig. 5 and 6A). An ANOVA comparing the mean voltages of deviant and standard responses in the latency window around the peak of this deflection (21 to 27 ms) over the electrodes Fz, FCz, Cz, FC3 and FC4 was significant for the stimulus type (F(1,22) = 10.686, p<.01). The difference consisted in a more negative response to deviants in comparison to standards. *Post-hoc* tests resulted in significant differences (Fig. 5) at the electrodes Fz (t(22) = −3.342, p<.05), FCz (t(22) =  −3.385, p<.05) and FC3 (t(22) =  −4.178, p<.01). Testing for differences in standard and control mean voltages (21 to 27 ms) over the electrodes Fz, FCz, Cz, FC3 and FC4 resulted in a significantly stronger response to the control than to the standard stimulus (Fig. 6C,D; F(1,22) = 9.727, p<.01). This difference was significant at Fz (t(22) =  −3.610, p<.05), FCz (t(22) =  −3.154, p<.05) and FC3 (t(22) =  −3.385, p<.05). No significant differences were found for deviant and control responses neither at latencies of the deviant-standard difference waveform nor at latencies of the deviant-control difference waveform (17 to 23 ms; Fig. 6B). Comparison of peak latencies revealed a significant difference for the Na peak latency between standards and controls at electrode Fz (z = −2.801, p<.05) with the control showing longer latencies (Fig. 6C).

A small MMN was elicited, that is the response to deviants was more negative than the standard response with a maximal difference at 173 ms after stimulus onset at the electrode FCz. The comparison of mean amplitudes (158−188 ms) at the electrode FCz revealed a significance (t(22) = −2.130, p<.05). Mean amplitudes of deviant and control stimuli, however, did not differ significantly from each other.

## Discussion

The objective of this study was to investigate whether MMN-like deviance-related modulations in response to intensity deviants were present in the MLR and ABR of the human AEP. The main finding was that at the transition from the Na to the Pa component of the MLR, at circa 24 ms from stimulus onset, click sounds of lower intensity that occurred in the role of deviants elicited a negative deflection compared to click sounds with the same physical intensity occurring in the role of standards.

MMN can be elicited pre-attentively by louder as well as by softer intensity deviants [2], [25], and peaks at approx. 200 ms after stimulus onset [25]. Our results suggest that in addition to intensity MMN, the detection of intensity deviants is reflected at much shorter latencies in the time range of the MLR, at the transition of the Na to the Pa component. The generators of the Na component are suggested to lie in the primary auditory cortex and the Pa component possibly has several sources in the primary, belt and parabelt regions [20−21], . This finding for intensity deviants is in agreement with recently reported modulations of the MLR triggered by frequency [Bibr pone.0028522-Grimm1] [9−10] and location [Bibr pone.0028522-Sonnadara1] [11−12] deviants. On the one hand, Grimm et al. [9] reported that frequency deviants elicited a more negative Nb component than standard and control stimuli of a controlled oddball paradigm. Given that the AEPs for clicks presented at 40 dB SL recorded in our study only revealed the components Na and Pa, specific comparisons to Grimm et al. 's results cannot be drawn and the possible existence of deviance-related modulations in the time range of the Nb and Pb components triggered by deviant intensities cannot be ruled out. On the other hand, an enhanced Pa component in response to broadband noise frequency deviants of a controlled oddball experiment was observed by Slabu et al. [10]. Furthermore, concerning sound location, it was shown that sounds presented from an infrequent location elicit an enhanced Na component [Bibr pone.0028522-Sonnadara1] [11−12]. Thus, the processing of location and intensity deviants appears to be reflected at earlier latencies than the processing of frequency deviants.

Besides fast deviance-related modulations in the MLR, deviant detection at single- and multi-unit levels beginning at latencies of about 20 ms [Bibr pone.0028522-Yu1] [6−7] is supported by several animal studies. These studies reported neurons in the auditory regions of the midbrain, thalamus and cortex exhibiting strong SSA to repetitive stimuli and restoration of their firing rates when a deviant stimulus occurred. This phenomenon has been mainly observed for frequency deviants [6−7], , but in a handful of studies, the processing of intensity deviants was tested as well. Ulanovsky et al. [16] found that in the cat primary auditory cortex, louder intensity deviants triggered an increased firing rate compared to standard stimuli, but softer did not (cf. discussion of Farley and colleagues [24]). This was confirmed by Farley et al. [24], who recorded from the primary auditory cortex of the awake rat. According to Pérez-González et al. [13], already neurons in the IC of the rat exhibit SSA and an enhancement in the firing rate in response to intensity deviants. Furthermore, in the midbrain of the barn owl, SSA and enhanced response to deviant intensities was detected [19]. Importantly, the data of this later study suggests that a release from SSA is not only triggered by a louder novel stimulus, but also by a weaker stimulus following a sequence of 10 repetitive stimuli. However, at the cellular level, results regarding softer intensity deviants are contradictory and more detailed investigation concerning this matter is required.

The relationship between MMN and SSA, as two measures of the enhanced responsiveness to deviant stimuli, has not been clarified yet and there are constraints in comparing the effects of intracranial cellular recordings to scalp evoked potentials, as the latter are a composed signal reflecting the concurrent activity of many different neural populations. A bridge between cellular responses and scalp-measured responses is provided by evoked local field potentials (*e*LFPs), which were recorded simultaneously with cellular responses in a frequency oddball paradigm from the rat auditory cortex [24], and more specifically, from the rat primary auditory cortex [18]. Similar to the increased response measured at the cellular level, the first negative deflection of the *e*LFP showed an enhanced response to deviants compared to standards [18], [24], with the difference wave peaking at approx. 25 ms after stimulus onset [18].

A significant difference between the deviant and control ERP was not observed in this study. The control condition was implemented because there might exist an amplitopicity in the auditory cortex [Bibr pone.0028522-Bilecen1] [34−35], that is, a systematical encoding of intensity similar to the tonotopic organization. This would imply that an enhanced response to the deviant compared to the standard could be explained by refractoriness of the neurons responding to the standard stimulus and the recruitment of “fresh” neurons when responding to the deviant. However, the control condition, as it is applied in this study, may overcontrol for refractoriness [Bibr pone.0028522-Lazar1] [36−37], because the neurons that respond to the deviant stimuli might be more refractory than the neurons responding to the control stimuli, as the constant activation elicited by the standard stimulus could also fatigue the neighboring neural population responding to the deviant. This effect has been demonstrated by Taaseh et al. [17] who examined the detection of frequency deviants by means of *e*LFP and multi-unit activity recordings in the rat auditory cortex. They showed that, on the one hand, comparison of the deviant response and the response to a control stimulus embedded in a control condition, where the frequency separation between the control stimuli is narrower than the one between deviant and standard stimuli in the oddball condition, results in a stronger neural response to the deviant than to the control stimulus. On the other hand, responses to the deviant are not stronger than responses to a control stimulus that is embedded in a control condition where the frequency separation between the different control stimuli is the same as it is between deviant and standard stimulus in the oddball condition. Especially in the experimental design used in the present study, it is likely that the neural population responding to the softer deviant is also activated during the presentation of the louder standard stimulus since louder stimuli activate a wider neural region in the auditory cortex than softer stimuli [34]. This might dispel the notion that fresh neurons are recruited during presentation of the deviant stimulus when it is softer in intensity than the standard. Likewise it is suggested that when using a softer deviant, the MMN comprises no activation from the N1 generator process, as the N1 generator process should be attenuated compared to its activation by the louder standard stimulus [32]. Assuming this, the enhanced MLR response to softer deviants observed in our study could be interpreted as the reflection of a sensory novelty processing rather than a release from refractoriness. Additionally, the fact that intensity deviants elicited a differential response compared to the standards not at the peak of any particular MLR waveform, but at the transition of the Na to the Pa waveform, provides further support in favor of a “genuine” deviance-related response. One may speculate that such an additional neuroelectric activity might be elicited by a specific neural population showing an enhanced activation (in terms of a release from SSA) at a latency that is independent or delayed compared to the neuronal population's activity giving rise to the specific MLR waveforms.

The consistent and reliable analysis of sounds reflected by AEP components is nicely expressed in the ABRs to the stimuli of the control condition. Sound intensity is consistently modulating Wave V, which is systematically increasing in amplitude and systematically decreasing in latency with increasing stimulation intensity as it has already been shown in seminal studies ([Bibr pone.0028522-Elberling1] [38−39]; for a review, see [40]). Nonetheless, to the authors' opinion, it is worth depicting this relationship using up-to-date plotting techniques here, most importantly to show that it is possible to obtain excellent data quality by recoding only 1,248 trials per stimulus type. This is revealed by the flat baseline and the low threshold of Wave V (which is already elicited from stimulation with 10 to 20 dB SL on).

Wave V of the ABR showed no differences in mean amplitude or latency between the stimulus types deviant, standard and control, which is consistent with the results of the study by Slabu et al. [10] on broadband noise frequency deviants. The absence of a deviance-related modulation in the ABR was expected, as it reflects the first volley of activations at auditory stations up to the IC [41]. Additionally, it is characteristically consistent and analyzes sounds quickly and reliably [40]. The subcortical neurons that exhibit sensitivity to deviant auditory stimuli in animals are mostly located in non-primary subdivisions of the thalamus and the IC [7], . These non-primary portions are innervated by dense top-down projections [Bibr pone.0028522-Malmierca2] [42−43] whereas primary regions are part of the afferent pathway [42], [44]. ABRs, however, reflect activity from the afferent auditory pathway [40], which might explain why no deviance-related modulations in the ABR have been found in the present and previous studies.

There was only a small MMN elicited for comparison of deviant and standard; and no difference was observed for comparison of deviant and control. This is probably due to characteristics of the sequences' design, which were tailored to record ABRs, MLRs and LLR responses simultaneously. First, click sounds, which were used to elicit ABR and MLR components, are not optimal to elicit MMN [31]. Second, the usage of a rather short and random SOA with a mean of 300 ms might have led to the small MMN because the temporal probability of deviants increases with decreasing SOA. This leads to a decline of MMN amplitude [45].

The feature which differentiates the deviant from the standard stimuli in this study was sound intensity. Regarding the perception of sound intensity it has to be considered that the perceived intensity of a sound depends on the sound duration, because a temporal integration of loudness over the first hundreds of milliseconds takes place [Bibr pone.0028522-Verhey1] [46−47]. As the used stimuli in this study were click sounds with a very short duration of 100 µs, temporal integration of loudness does not contribute to their loudness perception. This raises the question if the detection of intensity deviants for stimuli with a longer duration is based on an additional, later comparison mechanism which takes into account the temporal integration of loudness and would only be reflected at later latencies of the AEP that is by MMN.

In summary, our results confirm the idea that the detection of deviant and contextual novel sounds is a pervasive property of the auditory system. It has so far been shown that frequency, location and intensity deviants are automatically detected in the first 40 ms from stimulus onset and additionally in the later processes reflected by MMN. The existence of auditory novelty detection at early stages is supported by animal studies, which suggest that even at the subcortical level, stimulus feature changes are encoded by single neurons. The distinction between standard and novel or deviant sounds is an essential cognitive ability as it is important e.g. for auditory attention and the perception of sounds that signal a harmful situation. Therefore, the exact understanding of the novelty detection pathway is not only an important step in the exploration of the cognitive system, but can also contribute to successful diagnosis of cognitive dysfunctions and the development of their treatments.
